# Is There a Better Day of the Week to Administer Semaglutide or Tirzepatide? A Systematic Literature Review

**DOI:** 10.3390/medicina62081536

**Published:** 2026-08-10

**Authors:** Sandro La Vignera, Rosita A. Condorelli

**Affiliations:** Department of Clinical and Experimental Medicine, University of Catania, Via S. Sofia 78, 95123 Catania, Italy; rosita.condorelli@unict.it

**Keywords:** GLP-1 receptor agonists, semaglutide, tirzepatide, administration timing, medication adherence, type 2 diabetes, obesity, chronotherapy

## Abstract

*Background and Objectives*: Glucagon-like peptide-1 receptor agonists (GLP-1 RAs), including semaglutide and the dual GIP/GLP-1 receptor agonist tirzepatide, have transformed the management of type 2 diabetes mellitus (T2DM) and obesity through once-weekly subcutaneous administration. While their efficacy and safety are well-established, the potential impact of administration timing—specifically, the day of the week—on clinical outcomes, adherence, and tolerability remains unexplored. Understanding whether specific days optimize therapeutic outcomes could inform personalized dosing strategies and improve long-term treatment success. *Materials and Methods*: We conducted a systematic literature review following PRISMA 2020 guidelines to identify studies examining the effect of weekly administration timing of semaglutide or tirzepatide on clinical outcomes in adults with T2DM or obesity. Comprehensive searches were performed across SciSpace (Deep Search, Basic Search, Full Text Search), Google Scholar, and PubMed databases through June 2026. Studies were screened using a two-stage process (abstract and full-text screening) with predefined PICO-based inclusion criteria. Data extraction focused on study design, population characteristics, administration timing, glycemic control, weight loss, adherence, and adverse events. *Results*: From 1471 identified records, 1000 unique papers underwent screening after deduplication and trimming. Three studies met inclusion criteria: the SUSTAIN 4 randomized controlled trial evaluating once-weekly semaglutide, a retrospective case series examining alternate-day oral semaglutide dosing, and an expert panel discussion on flexible dosing schedules. No studies directly compared different days of the week for injectable semaglutide or tirzepatide administration. Available evidence suggests that flexible dosing schedules may support adherence and tolerability without compromising glycemic control, though this suggestion derives primarily from indirect evidence and expert opinion rather than direct comparative clinical studies; direct comparative data on day-of-week effects are absent. *Conclusions*: Current evidence does not support a specific optimal day of the week for semaglutide or tirzepatide administration. The limited literature emphasizes flexible dosing schedules tailored to individual patient preferences and lifestyles to maximize adherence. Future prospective studies are needed to systematically evaluate whether administration timing influences clinical outcomes in real-world settings.

## 1. Introduction

Type 2 diabetes mellitus (T2DM) and obesity represent interconnected global health challenges affecting hundreds of millions of individuals worldwide and contributing substantially to cardiovascular morbidity, mortality, and healthcare expenditure. The therapeutic landscape for these metabolic disorders has been revolutionized by glucagon-like peptide-1 receptor agonists (GLP-1 RAs), a class of medications that enhance glucose-dependent insulin secretion, suppress glucagon release, delay gastric emptying, and promote satiety through central nervous system mechanisms [1]. Among the most potent and widely prescribed agents in this class are semaglutide, a selective GLP-1 RA, and tirzepatide, a novel dual glucose-dependent insulinotropic polypeptide (GIP) and GLP-1 receptor agonist. Both medications offer once-weekly subcutaneous administration, providing a convenient alternative to daily injectable therapies and potentially improving patient acceptance and long-term adherence.

### 1.1. Pharmacology of Semaglutide and Tirzepatide

Semaglutide is a long-acting GLP-1 RA with approximately 94% structural homology to native human GLP-1. Its extended half-life of approximately 7 days is achieved through albumin binding and fatty acid modification, enabling once-weekly subcutaneous dosing [2]. In the SUSTAIN clinical trial program, semaglutide demonstrated superior reductions in HbA1c and body weight compared to multiple comparators, including insulin glargine, sitagliptin, dulaglutide, and exenatide once weekly [1,3,4]. Tirzepatide, a dual GIP/GLP-1 receptor agonist with a half-life of approximately 5 days [5], has demonstrated even greater efficacy in the SURPASS trial series, with HbA1c reductions of up to 2.3% and weight loss exceeding 20% in some patient populations [6,7,8]. The complementary mechanisms of GIP and GLP-1 receptor activation may synergistically enhance insulin secretion, glucagon suppression, and energy expenditure.

Both agents are associated with gastrointestinal adverse events, particularly nausea, vomiting, and diarrhea, which are most pronounced during dose escalation and tend to diminish over time. These adverse effects represent the primary reason for treatment discontinuation and may be influenced by the timing of administration relative to meals, daily activities, and individual physiological rhythms.

### 1.2. Rationale for Weekly Dosing and Administration Timing

The once-weekly dosing regimen of semaglutide and tirzepatide simplifies treatment and has the potential to enhance medication adherence and persistence compared to daily injectable therapies [9]. However, an important clinical question remains largely unexplored: does the specific day of the week chosen for administration influence clinical outcomes, adherence, or adverse events? Patients and clinicians often select injection days based on convenience, lifestyle factors, or personal preferences, but there is limited evidence to guide these decisions.

Theoretical considerations suggest that administration timing could affect treatment outcomes through several mechanisms. Weekend dosing might align better with patients’ schedules, potentially improving adherence, while weekday dosing could facilitate closer monitoring and support from healthcare providers. Additionally, the pharmacokinetic profiles of these agents might interact with weekly patterns of diet, physical activity, and stress, potentially influencing glycemic variability and weight loss trajectories.

### 1.3. Chronopharmacology and Circadian Biology

Circadian rhythms govern numerous physiological processes relevant to the pharmacodynamics of GLP-1 RAs and dual agonists, including insulin secretion, glucose metabolism, appetite regulation, gastric motility, and energy expenditure. The emerging field of chronopharmacology explores how the timing of drug administration relative to endogenous biological rhythms influences therapeutic efficacy and tolerability. While circadian effects have been demonstrated for several antidiabetic agents, including insulin and metformin, the interaction between day-of-week administration and the pharmacodynamics of once-weekly agents such as semaglutide and tirzepatide has not been systematically investigated.

Given the increasing use of these agents in clinical practice and the growing emphasis on personalized medicine, a systematic evaluation of the evidence regarding administration timing is warranted. Understanding whether specific days of the week optimize clinical outcomes or adherence could inform evidence-based recommendations and support shared decision-making between patients and healthcare providers.

### 1.4. Objectives

The primary objective of this systematic review is to synthesize the available evidence on whether the specific day of the week for administering semaglutide or tirzepatide affects glycemic control, weight loss outcomes, and patient adherence in adults with T2DM or obesity. Secondary objectives include examining the impact of administration timing on adverse events, patient-reported outcomes, and treatment satisfaction. By systematically reviewing the literature, we aim to identify knowledge gaps and provide recommendations for future research and clinical practice.

## 2. Materials and Methods

### 2.1. Protocol and Registration

This systematic review was conducted in accordance with the Preferred Reporting Items for Systematic Reviews and Meta-Analyses (PRISMA) 2020 guidelines. The review protocol was not registered prospectively. This absence of prospective registration constitutes a methodological limitation, as it reduces transparency and increases the theoretical risk of selective outcome reporting.

### 2.2. PICO Framework

The research question was structured using the PICO framework:

**Population (P):** Adults (≥18 years) diagnosed with type 2 diabetes mellitus (T2DM) or obesity/overweight.

**Intervention (I):** Specific day of the week chosen for the administration of semaglutide or tirzepatide (subcutaneous or oral formulations).

**Comparison (C):** Other days of the week or no specific day preference (flexible or patient-chosen dosing schedule).

**Outcomes (O):** Glycemic control (HbA1c, fasting plasma glucose, time-in-range), body weight and weight loss, medication adherence and persistence, adverse events (particularly gastrointestinal), and patient-reported outcomes.

### 2.3. Search Strategy

A comprehensive literature search was performed across multiple databases and platforms to ensure maximum coverage of the relevant literature. The search was conducted through 15 June 2026, with no language restrictions. All search queries are documented in the Supplementary Materials. Clinical trial registries (ClinicalTrials.gov, WHO ICTRP, EU Clinical Trials Register) and grey literature sources were not systematically searched, representing an acknowledged limitation of the search strategy. The following databases were searched:

• SciSpace Deep Search: semantic search using natural language queries focused on administration timing, day of the week, and clinical outcomes (n = 230 papers).
• SciSpace Basic Search: two structured queries combining GLP-1 RA terminology with dosing schedule and adherence terms (n = 200 papers).
• SciSpace Full Text Search: three full-text queries targeting specific terminology related to administration timing and day-of-week effects (n = 300 papers).
• Google Scholar: three comprehensive queries with auto-pagination, combining terms including “Semaglutide,” “Tirzepatide,” “GLP-1 receptor agonist,” “administration timing,” “dosing schedule,” “day of week,” “adherence,” “glycemic control,” “weight loss,” “type 2 diabetes,” and “obesity” (n = 219 papers).
• PubMed: three advanced queries using Medical Subject Headings (MeSH) terms and Title/Abstract field tags, focusing on semaglutide, tirzepatide, administration timing, clinical outcomes, and target populations (n = 522 papers).

### 2.4. Eligibility Criteria

**Inclusion criteria:**

• Adult patients (≥18 years) diagnosed with T2DM or obesity/overweight.
• Studies investigating semaglutide or tirzepatide (subcutaneous or oral formulations) as the primary intervention.
• Studies analyzing or comparing the timing of administration based on specific days of the week, day preferences, or flexible dosing schedules.
• Studies reporting on glycemic control, weight loss, medication adherence, adverse events, or patient-reported outcomes.

**Exclusion criteria:**

• Preclinical studies, animal models, cell cultures, or in vitro studies.
• Studies focusing primarily on other GLP-1 receptor agonists (e.g., liraglutide, dulaglutide, exenatide) without comparison to semaglutide or tirzepatide.
• Studies evaluating general efficacy or safety of semaglutide or tirzepatide without any analysis of day-of-week or administration timing effects.

### 2.5. Screening Process

Screening was conducted in two stages. In Stage 1 (Abstract Screening), all 1000 unique records (after deduplication) were evaluated against predefined PICO-based criteria using an AI-assisted methodology in which each record was scored against five predefined PICO-based criteria (population appropriateness, intervention relevance, comparison group, outcome reporting, and study design acceptability), with equal weight (1 point each) on a scale of 1 to 5. Papers scoring ≥ 4.0 (scale 1–5) advanced to full-text review. In Stage 2 (Full-Text Screening), retrieved full texts were assessed in detail; a higher threshold (score ≥ 4.5) was applied to ensure inclusion of only clearly relevant studies.

### 2.6. Data Extraction

Data were extracted systematically from all included studies using a standardized form capturing: study design; population characteristics (sample size, age, sex, diagnosis, baseline HbA1c, body weight, BMI); administration timing details (day of week, fixed vs. flexible schedule); primary outcomes (HbA1c, fasting plasma glucose, weight change); adherence metrics (adherence rates, persistence, patient satisfaction); and adverse events (incidence and severity of gastrointestinal side effects, injection site reactions).

### 2.7. Quality Assessment

The methodological quality of included studies was assessed using appropriate tools: the Cochrane Risk of Bias tool (RoB 2.0) for the randomized controlled trial, the Newcastle-Ottawa Scale (NOS) for the observational case series, and the AGREE II instrument for the expert panel discussion. Given the small number of included studies and the heterogeneity in study designs, a narrative synthesis was performed rather than a meta-analysis. The AGREE II instrument, applied to the expert panel discussion by Candido et al. (2024), was originally developed and validated for clinical practice guidelines [10]; its application here represents an acknowledged methodological deviation, adopted as the most appropriate available tool for this type of document.

## 3. Results

### 3.1. Study Selection

**Figure 1:**
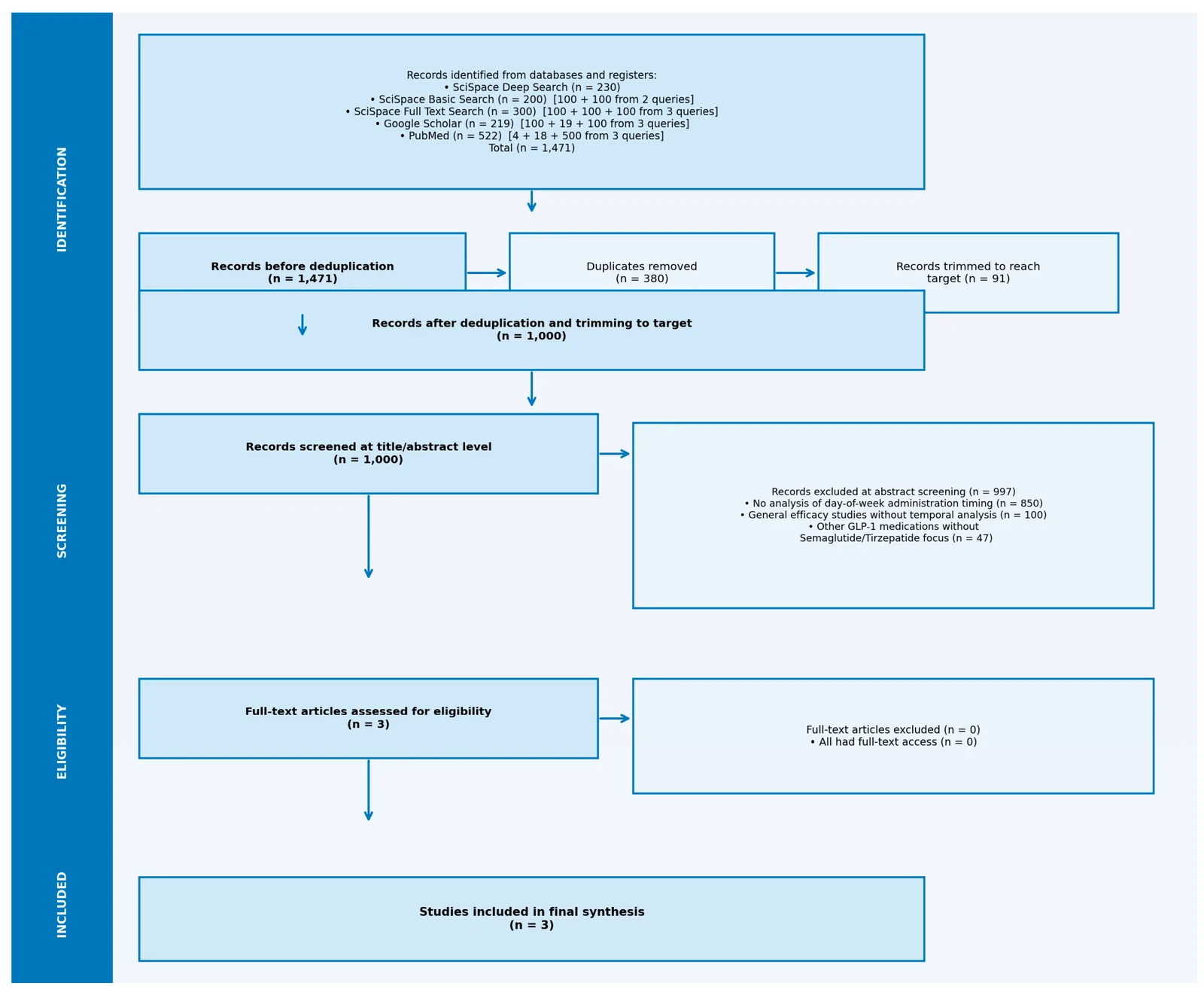
PRISMA 2020 flow diagram showing the study selection process.

The systematic search identified 1471 records across all databases. After removing 380 duplicate records and trimming an additional 91 records to reach the screening target, all 1000 records underwent abstract screening. Of these, 997 papers were excluded at the abstract screening stage: 850 did not analyze administration timing or day-of-week effects; 100 evaluated general efficacy without temporal analysis; and 47 focused on other GLP-1 receptor agonists. Three papers met the inclusion criteria at abstract screening and proceeded to full-text assessment. All three passed full-text screening and were included in the qualitative synthesis. No additional studies were identified through reference list screening or citation searching. The study selection process is summarized in the PRISMA 2020 flow diagram (Figure 1).

### 3.2. Characteristics of Included Studies

The characteristics of the three included studies are summarized in Table 1. The included studies comprised one randomized controlled trial (SUSTAIN 4), one retrospective observational case series, and one expert panel discussion. None of the studies was specifically designed to compare different days of the week for semaglutide or tirzepatide administration; however, each provided relevant evidence regarding dosing flexibility, administration timing, or adherence. A formal meta-analysis was not feasible given the heterogeneity in study designs (RCT, uncontrolled retrospective case series, and expert panel discussion) and populations across the included studies.

#### 3.2.1. SUSTAIN 4 Trial (Aroda et al., 2017) [1]

The SUSTAIN 4 trial was a 30-week, randomized, open-label, parallel-group, multicentre, multinational, phase 3a trial comparing once-weekly subcutaneous semaglutide (0.5 mg and 1.0 mg) to once-daily insulin glargine as add-on therapy to metformin (with or without sulfonylureas) in 1089 insulin-naive patients with T2DM [1]. Semaglutide demonstrated statistically significant superiority over insulin glargine in reducing HbA1c (estimated treatment difference: −1.21% for semaglutide 1.0 mg) and body weight (estimated treatment difference: −5.94 kg for semaglutide 1.0 mg). Patients were instructed to administer semaglutide once weekly, with the choice of day left to individual preference; no subgroup analyses based on the day of administration were reported. This trial provides the highest level of evidence for once-weekly semaglutide efficacy but does not address the specific clinical question of day-of-week effects. Of note, patients were free to choose their preferred day of injection, and no subgroup analysis by day of administration was conducted or reported in the trial.

#### 3.2.2. Alternate-Day Oral Semaglutide Case Series (RoyChaudhuri et al., 2023) [11]

RoyChaudhuri and colleagues presented a retrospective case series of 10 patients with T2DM who adopted an alternate-day dosing regimen for oral semaglutide (14 mg) to minimize gastrointestinal side effects [11]. Ambulatory glucose profile (AGP) data were compared between days-on-drug and days-off-drug. The analysis revealed no significant differences in time-in-range (TIR), time-above-range (TAR), or time-below-range (TBR) between the two types of days. This finding suggests that the pharmacodynamic glucose-lowering effects of oral semaglutide may persist beyond the immediate post-dose period, supporting the feasibility of flexible dosing schedules. While this study provides preliminary evidence that dosing flexibility may maintain glycemic control while improving tolerability, key limitations must be acknowledged: the very small sample size (n = 10) precludes generalizability, alternate-day oral semaglutide is not an approved dosing regimen, and the absence of a control group limits causal inference.

#### 3.2.3. Expert Panel on Flexible Dosing (Candido et al., 2024) [10]

Candido and colleagues convened an Italian expert panel to evaluate the suitability and usefulness of flexible dosing timing for oral semaglutide in clinical practice [10]. The panel reviewed anecdotal evidence from a small case series and real-world database, concluding, based on expert opinion and limited observational data rather than controlled clinical trial evidence, that flexibility in the timing of oral semaglutide administration may support adherence and persistence without compromising glycemic control or weight loss outcomes. The experts emphasized the importance of individualizing dosing schedules based on patient preferences, lifestyle factors, and tolerability. These conclusions should be treated as hypothesis-generating expert consensus statements rather than evidence-based clinical recommendations. The correct pagination for this reference is pages 1963–1977 of volume 15.

### 3.3. Synthesis of Evidence

#### 3.3.1. Administration Timing and Day of the Week

None of the included studies directly compared different days of the week for semaglutide or tirzepatide administration in terms of clinical outcomes, adherence, or adverse events. The SUSTAIN 4 trial allowed patients to choose their preferred day for once-weekly semaglutide injection but did not analyze outcomes based on the selected day [1]. The case series by RoyChaudhuri et al. examined alternate-day dosing of oral semaglutide but did not evaluate specific days of the week [11]. The expert panel discussion by Candido et al. emphasized flexible dosing schedules but did not provide empirical data comparing specific days [10]. The complete absence of studies directly addressing this question constitutes the primary and most important finding of this systematic review; the absence of evidence should not be interpreted as evidence of clinical equivalence.

#### 3.3.2. Glycemic Control

The SUSTAIN 4 trial demonstrated that once-weekly semaglutide significantly reduced HbA1c regardless of the administration day chosen [1]. The case series by RoyChaudhuri et al. found that alternate-day oral semaglutide maintained comparable glycemic control (TIR, TAR, TBR) on both days-on-drug and days-off-drug, suggesting glucose-lowering effects persist beyond the immediate post-dose period [11]. The expert panel noted that flexible dosing schedules do not appear to compromise glycemic control [10]. In the SUSTAIN 4 trial, the estimated treatment difference in HbA1c reduction versus insulin glargine was −1.21% for semaglutide 1.0 mg and −0.83% for semaglutide 0.5 mg (both *p* < 0.0001); mean baseline HbA1c was approximately 8.1% across groups.

#### 3.3.3. Weight Loss Outcomes

The SUSTAIN 4 trial reported significant weight loss with once-weekly semaglutide compared to insulin glargine, but no subgroup analyses based on administration day were conducted [1]. Neither the case series by RoyChaudhuri et al. nor the expert panel by Candido et al. provided specific weight loss data in relation to administration timing [10,11]. In the SUSTAIN 4 trial, the estimated treatment difference in body weight versus insulin glargine was −5.94 kg for semaglutide 1.0 mg and −3.47 kg for semaglutide 0.5 mg (both *p* < 0.0001); these results are not stratified by day of administration.

#### 3.3.4. Adherence and Patient Satisfaction

The expert panel discussion by Candido et al. highlighted that flexible dosing schedules may enhance adherence and persistence by allowing patients to align administration with personal routines and preferences [10]. The case series by RoyChaudhuri et al. reported that alternate-day dosing was adopted to improve tolerability, which could indirectly support adherence [11]. However, neither study provided quantitative adherence data or patient satisfaction scores. It is important to note that the suggestion that flexible dosing may enhance adherence is based solely on expert opinion and indirect inference; no quantitative adherence measures were reported in any included study in relation to administration timing or day-of-week selection.

#### 3.3.5. Adverse Events

Gastrointestinal side effects—nausea, vomiting, diarrhea—are common with GLP-1 RAs and can affect treatment persistence. RoyChaudhuri et al. reported that alternate-day oral semaglutide was adopted to minimize gastrointestinal symptoms [11]. Candido et al. noted that individualized dosing schedules can help manage adverse events and support continued therapy [10]. No studies directly compared adverse event incidence based on the specific day of administration. The suggestion that individualized dosing schedules can help manage gastrointestinal adverse events is derived from expert opinion and pharmacokinetic reasoning, not from head-to-head comparative clinical data.

## 4. Discussion

### 4.1. Summary of Findings

This systematic review aimed to synthesize the available evidence on whether the specific day of the week for administering semaglutide or tirzepatide affects glycemic control, weight loss, patient adherence, adverse events, or patient-reported outcomes in adults with T2DM or obesity. Despite a comprehensive search screening 1000 unique records, only three studies met the inclusion criteria, and none directly compared different days of the week for administration. This finding itself constitutes an important result, highlighting a significant and clinically meaningful gap in the literature. This review therefore constitutes primarily an evidence-gap analysis rather than a traditional systematic synthesis of existing direct evidence; any clinical inferences drawn are explicitly grounded in indirect evidence and acknowledged as such.

The pharmacokinetic profiles of semaglutide (half-life ~7 days) and tirzepatide (half-life ~5 days) result in relatively stable plasma concentrations throughout the week after steady state is achieved, theoretically supporting the feasibility of flexible dosing without compromising efficacy. The available evidence, while indirect, is consistent with this pharmacological rationale: flexible dosing schedules appear to maintain glycemic control and may improve tolerability and adherence, based primarily on indirect evidence and expert opinion rather than direct comparative data [10,11].

### 4.2. Chronopharmacology and Clinical Implications

The concept of chronopharmacology—the study of how biological rhythms influence drug pharmacokinetics and pharmacodynamics—is increasingly recognized as relevant to metabolic disease management. Circadian rhythms govern insulin secretion, glucose homeostasis, gastric emptying, and appetite regulation. For once-daily medications, the time of administration relative to these rhythms can significantly influence efficacy and tolerability. However, for once-weekly agents such as semaglutide and tirzepatide, the long half-lives and sustained pharmacodynamic effects may attenuate circadian influences on drug action, making the specific day of the week less critical than the consistency of weekly administration. It should be emphasized that these chronopharmacological considerations are presented as a theoretical scientific framework for future research, not as evidence-based conclusions derived from the included studies; no included study examined circadian influences on semaglutide or tirzepatide pharmacokinetics or pharmacodynamics in relation to day-of-week administration.

Nevertheless, indirect circadian effects may still be relevant. For example, if administration on weekdays is associated with greater dietary control or physical activity compared to weekends—or if weekend dosing allows patients more time to manage gastrointestinal side effects without work-related interference—these behavioral factors could influence treatment outcomes. Future research should explore these potential interactions.

### 4.3. Implications for Clinical Practice

Based on the available evidence, clinicians should engage patients in shared decision-making when selecting the day of the week for semaglutide or tirzepatide administration. Key considerations include: (1) work and social schedules—patients may prefer to administer on a day when they can manage potential side effects at home; (2) healthcare access—weekday dosing may facilitate closer monitoring; (3) routine and memory aids—selecting a consistent, memorable day (e.g., linked to a weekly routine) supports adherence; and (4) tolerability—patients experiencing gastrointestinal side effects may benefit from adjusting the administration day to align with lower-activity periods.

Based on indirect evidence and expert consensus (approved prescribing information, pharmacokinetic rationale, and expert consensus opinion) rather than direct comparative clinical studies, flexible dosing schedules may support adherence and tolerability. Healthcare providers should emphasize consistency in weekly administration regardless of the specific day chosen, and should proactively counsel patients on the management of gastrointestinal adverse events.

### 4.4. Limitations

This systematic review has several important limitations. First, only three studies met the inclusion criteria, and none directly compared different days of the week for administration, severely limiting the strength of conclusions. Second, the included studies varied considerably in design (RCT, case series, expert panel), population, and outcomes, precluding meta-analysis. Third, no studies examining tirzepatide administration timing were identified, reflecting the relatively recent introduction of this agent. Fourth, the review relied on published literature and did not include unpublished studies, conference abstracts, or grey literature. Fifth, the search strategy may not have captured all relevant studies addressing dosing flexibility without explicitly mentioning day-of-week effects. Additional limitations include: the review protocol was not prospectively registered, reducing methodological transparency; the AI-assisted abstract screening is not fully reproducible by independent reviewers due to proprietary algorithm parameters; and the search did not include clinical trial registries or grey literature sources. Finally, as the primary conclusion of this review concerns the identification of a significant gap in the existing literature rather than the synthesis of direct comparative evidence, it is important to acknowledge additional inherent limitations. A gap-identification conclusion is subject to potential language bias, as only English-language studies were included; the possibility that relevant studies were conducted but remain unpublished or indexed in databases not searched; and the fundamental constraint that an absence of published evidence cannot be equated with an absence of a clinical effect. These limitations further reinforce the necessity of prospective, specifically designed studies to directly address the impact of day-of-week administration timing on clinical outcomes of semaglutide and tirzepatide.

### 4.5. Future Research Directions

The findings of this review highlight several important directions for future research. Prospective randomized or observational studies are needed to directly compare different days of the week for semaglutide and tirzepatide administration, assessing glycemic control, weight loss, adherence, adverse events, and patient-reported outcomes over extended follow-up periods. Research should also explore the mechanistic pathways by which administration timing might influence outcomes, including interactions with circadian rhythms, dietary patterns, physical activity, and stress. Real-world evidence studies using large databases or registries could provide valuable insights into patterns of administration timing and their clinical associations. Finally, patient-reported outcomes, including quality of life, treatment satisfaction, and preferences for administration timing, should be systematically assessed to inform patient-centered dosing recommendations. Specifically, proposed study designs include: a prospective randomized crossover trial comparing weekday versus weekend administration of once-weekly semaglutide or tirzepatide; large-scale retrospective registry studies using real-world data; and analyses incorporating continuous glucose monitoring and validated patient-reported adherence measures.

## 5. Conclusions

This systematic review found no evidence to support a specific optimal day of the week for semaglutide or tirzepatide administration in adults with type 2 diabetes or obesity. The limited available literature, derived primarily from indirect evidence and expert opinion rather than direct comparative clinical studies, suggests that flexible dosing schedules tailored to individual patient preferences and lifestyles may support adherence and treatment persistence, though this has not been confirmed by direct comparative clinical evidence. Clinicians should engage patients in shared decision-making when selecting the day of administration, considering work schedules, social activities, and tolerability of adverse events. The pharmacokinetic profiles of semaglutide and tirzepatide—characterized by long half-lives and sustained pharmacodynamic effects—support the feasibility of flexible dosing without compromising glycemic control or weight loss outcomes. Future prospective studies are urgently needed to systematically evaluate the impact of administration timing on clinical outcomes, adherence, and patient-reported outcomes in real-world settings. It should be noted that the conclusion that no specific optimal day exists is a statement of evidence absence, not evidence of clinical equivalence; future studies may identify clinically meaningful day-of-week effects on outcomes. Importantly, the primary contribution of this systematic review is the identification of a significant gap in the existing literature: to date, no published clinical study has directly examined the effect of the specific day of the week of subcutaneous semaglutide or tirzepatide administration on clinical outcomes in patients with T2DM or obesity. This gap constitutes, in itself, a meaningful and actionable finding that provides a clear rationale and foundation for future prospective research specifically addressing this unresolved clinical question.

## Figures and Tables

**Table 1:** Characteristics of included studies.

Study	Design	Population	Sample Size	Intervention	Key Findings
Aroda et al., 2017 [1]	Randomized, open-label, parallel-group, multicentre, multinational, phase 3a trial (SUSTAIN 4)	Insulin-naive adults with T2DM inadequately controlled with metformin ± sulfonylureas	N = 1089	Once-weekly subcutaneous semaglutide (0.5 or 1.0 mg) vs. once-daily insulin glargine, 30 weeks	Superior HbA1c reduction and weight loss with semaglutide vs. insulin glargine. No analysis of day-of-week effects; injection day chosen by patient preference.
RoyChaudhuri et al., 2023 [11]	Retrospective observational case series	Adults with T2DM on oral semaglutide 14 mg	n = 10	Oral semaglutide 14 mg alternate-day dosing (to minimize GI side effects)	No significant difference in TIR, TAR, or TBR between days-on-drug and days-off-drug. Suggests pharmacodynamic effects persist beyond the day of administration.
Candido et al., 2024 [10]	Expert panel discussion	Adults with T2DM in Italian clinical practice	N/A	Oral semaglutide with flexible dosing timing (patient-preferred schedule)	Expert consensus supports flexible dosing timing to maximize adherence and tolerability. No compromise of glycemic control with flexible scheduling.

## Data Availability

All data supporting the findings of this study are available within the article. The search strategies and PRISMA flow diagram are included in the manuscript.

## Supplementary Materials

The following supporting information can be downloaded at: https://www.mdpi.com/article/10.3390/medicina62081536/s1; File S1: PRISMA 2020 Checklist [12].
