# Supplementary material for: Is There a Better Day of the Week to Administer Semaglutide or Tirzepatide? A Systematic Literature Review
Source: Medicina (Kaunas). 2026 Aug 10;62(8):1536. doi: 10.3390/medicina62081536 (PMC13515371; doi:10.3390/medicina62081536)
Supplement: Supplementary file 1 [file medicina-62-01536-s001.zip › medicina-4409855-supplementary.pdf]

PRISMA 2020 Checklist

Reference: Page MJ, McKenzie JE, Bossuyt PM, Boutron I, Hoffmann TC, Mulrow CD, et al. The PRISMA 2020 statement: an updated guideline for reporting systematic reviews. *BMJ* 2021;372:n71. doi: 10.1136/bmj.n71

Section 1 — TITLE

Item No.	Section/Topic	Checklist Item	Location in Manuscript
1	Title	Identify the report as a systematic review.	**Title page.** The title explicitly states: *"Is There a Better Day of the Week to Administer Semaglutide or Tirzepatide? A Systematic Literature Review."* The designation "Systematic Literature Review" is included in the title.

Section 2 — ABSTRACT

Item No.	Section/Topic	Checklist Item	Location in Manuscript
2	Abstract	Provide a structured summary including, as applicable: background; objectives; data sources; study eligibility criteria, participants, and interventions; study appraisal and synthesis methods; results; limitations; conclusions and implications of key findings; systematic review registration number.	**Abstract section.** A structured abstract is provided with the following headings: *Background* (rationale and gap), *Methods* (databases, screening process, PICO criteria, data extraction), *Results* (1,471 identified records; 1,000 screened; 3 included studies; narrative synthesis), and *Conclusions* (no optimal day identified; flexible dosing recommended; future research needed).

Section 3 — INTRODUCTION

Item No.	Section/Topic	Checklist Item	Location in Manuscript
3	Rationale	Describe the rationale for the review in the context of existing knowledge.	**Section 1 (Introduction)**, particularly subsections **1.1** (*Pharmacology of Semaglutide and Tirzepatide*), **1.2** (*Rationale for Weekly Dosing and Administration Timing*), and **1.3** (*Chronopharmacology and Circadian Biology*). The rationale is grounded in the pharmacokinetic profiles of both agents, the clinical question of whether administration timing influences outcomes, and the emerging field of

			chronopharmacology.
4	Objectives	Provide an explicit statement of the objective(s) or question(s) the review addresses.	**Section 1.4** (*Objectives*). The primary objective is explicitly stated: to synthesize evidence on whether the specific day of the week for administering semaglutide or tirzepatide affects glycemic control, weight loss, and patient adherence. Secondary objectives include examining adverse events, patient-reported outcomes, and treatment satisfaction.

Section 4 — METHODS

Item No.	Section/Topic	Checklist Item	Location in Manuscript
5	Eligibility criteria	Specify the inclusion and exclusion criteria for the review and how studies were grouped for the syntheses.	**Section 2.4** (*Eligibility Criteria*). Explicit inclusion criteria: adults (≥18 years) with T2DM or obesity; semaglutide or tirzepatide as primary intervention; studies analysing day-of-week or flexible dosing; outcomes including glycemic control, weight loss, adherence, or adverse events. Exclusion criteria: preclinical studies; studies on other GLP-1 RAs without semaglutide/tirzepatide comparison; studies evaluating general efficacy without administration timing analysis.
6	Information sources	Specify all databases, registers, websites, organisations, reference lists and other sources searched or consulted to identify studies. Specify the date when each source was last searched or consulted.	**Section 2.3** (*Search Strategy*). Five databases searched through **June 15, 2026**: SciSpace Deep Search (n = 230), SciSpace Basic Search (n = 200), SciSpace Full Text Search (n = 300), Google Scholar (n = 219), and PubMed (n = 522). No language restrictions were applied. All search queries are documented in the Supplementary Materials.
7	Search strategy	Present the full search strategies for all databases, registers and websites, including any filters and limits used.	**Section 2.3** (*Search Strategy*). The section describes the search approach for each database: semantic natural language queries (SciSpace Deep Search); structured queries combining GLP-1 RA terminology with dosing schedule and adherence terms (SciSpace Basic, Google Scholar); full-text queries targeting administration

			timing terminology (SciSpace Full Text); and advanced MeSH-based queries with Title/Abstract field tags (PubMed). Full search strings are documented in the Supplementary Materials.
8	Selection process	Specify the methods used to decide whether a study met the inclusion criteria of the review, including how many reviewers screened each record and each report retrieved, whether they worked independently, and if applicable, details of automation tools used in the process.	**Section 2.5** (*Screening Process*). Two-stage screening: **Stage 1** (Abstract Screening) — all 1,000 unique records evaluated against PICO-based criteria using an AI-assisted methodology with equal-weight scoring; papers scoring ≥4.0 (scale 1–5) advanced to full-text review. **Stage 2** (Full-Text Screening) — retrieved full texts assessed in detail with a higher threshold (score ≥4.5) to ensure inclusion of only clearly relevant studies.
9	Data collection process	Specify the methods used to collect data from reports, including how many reviewers collected data from each report, whether they worked independently, any processes for obtaining or confirming data from study investigators, and if applicable, details of automation tools used in the process.	**Section 2.6** (*Data Extraction*). Data were extracted systematically from all included studies using a **standardized extraction form** capturing: study design; population characteristics (sample size, age, sex, diagnosis, baseline HbA1c, body weight, BMI); administration timing details (day of week, fixed vs. flexible schedule); primary outcomes; adherence metrics; and adverse events.
10	Data items	List and define all outcomes for which data were sought. Specify whether all results that were compatible with each outcome domain in each study were sought (e.g. for all measures, time points, analyses), and if not, the methods used to decide which results to collect.	**Section 2.6** (*Data Extraction*). Pre-specified data items included: **glycemic control** (HbA1c, fasting plasma glucose, time-in-range [TIR], time-above-range [TAR], time-below-range [TBR]); **body weight and weight loss**; **medication adherence and persistence**; **adverse events** (incidence and severity of gastrointestinal side effects, injection site reactions); and **patient-reported outcomes**.
11	Study risk of bias assessment	Specify the methods used to assess risk of bias in the included studies, including details of the tool(s) used, how many reviewers assessed each study and whether they	**Section 2.7** (*Quality Assessment*). Risk of bias was assessed using appropriate design-specific tools: **Cochrane Risk of Bias tool (RoB 2.0)** for the randomized controlled trial (SUSTAIN 4); **Newcastle-Ottawa Scale (NOS)** for the

		worked independently, and if applicable, information on automation tools used in the process.	retrospective observational case series (RoyChaudhuri et al., 2023); and the **AGREE II instrument** for the expert panel discussion (Candido et al., 2024).
12	Effect measures	Specify for each outcome the effect measure(s) (e.g. risk ratio, mean difference) used in the synthesis or presentation of results.	**Section 2.6 and Section 3.3** (*Synthesis of Evidence*). Effect measures reported include: **HbA1c reduction** (estimated treatment difference in percentage points for the RCT); **body weight change** (kg); and **time-in-range metrics** (TIR, TAR, TBR as percentages). Given the heterogeneity of study designs, a narrative synthesis was performed and no pooled effect measures were calculated.
13	Synthesis methods	Describe the processes used to synthesise results of studies. If meta-analysis was performed, describe the model(s), method(s) to identify the presence and extent of statistical heterogeneity, and software package(s) used.	**Section 2.7** (*Quality Assessment*) and **Section 3.3** (*Synthesis of Evidence*). Given the small number of included studies (n = 3) and substantial heterogeneity in study designs (RCT, retrospective case series, expert panel), a **narrative synthesis** was performed. Meta-analysis was not feasible or appropriate. Results are synthesised narratively across five domains: administration timing, glycemic control, weight loss, adherence, and adverse events.
14	Reporting bias assessment	Describe any methods used to assess risk of reporting biases within or across studies.	**Section 4.4** (*Limitations*). The manuscript acknowledges that the review relied exclusively on **published literature** and did not include unpublished studies, conference abstracts, or grey literature, representing a potential source of reporting bias. This is explicitly identified as a limitation of the review.
15	Certainty assessment	Describe any methods used to assess certainty (or confidence) in the body of evidence for an outcome.	**Section 2.7** (*Quality Assessment*) and **Section 4.4** (*Limitations*). The certainty of evidence was assessed through design-specific quality appraisal tools (RoB 2.0, NOS, AGREE II). The limitations section explicitly notes that the small number of included studies and heterogeneity in designs substantially limit the strength of

			conclusions, precluding formal GRADE assessment.

Section 5 — RESULTS

Item No.	Section/Topic	Checklist Item	Location in Manuscript
16	Study selection	Describe the results of the search and selection process, including reasons for exclusion of potentially eligible studies considered in more detail, ideally using a flow diagram.	**Section 3.1** (*Study Selection*) and **Figure 1** (PRISMA 2020 flow diagram). From 1,471 identified records: 471 duplicates removed; 1,000 unique records screened; 997 excluded at abstract screening (850 did not analyse administration timing/day-of-week effects; 100 evaluated general efficacy without temporal analysis; 47 focused on other GLP-1 RAs); 3 assessed for full-text eligibility; 0 excluded at full-text stage; **3 studies included** in qualitative synthesis.
17	Study characteristics	Provide a citation for each included study and present its characteristics.	**Section 3.2** (*Characteristics of Included Studies*) and **Table 1**. Three studies are characterised: (1) SUSTAIN 4 RCT (Aroda et al., 2017; N = 1,089; once-weekly subcutaneous semaglutide vs. insulin glargine); (2) retrospective case series (RoyChaudhuri et al., 2023; n = 10; alternate-day oral semaglutide); (3) expert panel discussion (Candido et al., 2024; Italian clinical practice; flexible dosing timing).
18	Risk of bias in studies	Present assessments of risk of bias for each included study.	**Section 2.7** (*Quality Assessment*) and implicitly in **Sections 3.2.1–3.2.3.** Risk of bias was assessed using RoB 2.0 (SUSTAIN 4), NOS (RoyChaudhuri et al.), and AGREE II (Candido et al.). The narrative synthesis approach was adopted in recognition of the methodological heterogeneity and the limitations of the included study designs.
19	Results of individual studies	For all outcomes, present, for each study: (a) summary statistics for each group (where	**Sections 3.2.1–3.2.3** (individual study summaries). SUSTAIN 4: HbA1c reduction of −1.21% (semaglutide 1.0

		appropriate) and (b) an effect estimate and its precision (e.g. confidence interval), ideally using structured tables or plots.	mg vs. insulin glargine) and weight loss of −5.94 kg. RoyChaudhuri et al.: no significant differences in TIR, TAR, or TBR between days-on-drug and days-off-drug with alternate-day oral semaglutide. Candido et al.: expert consensus supporting flexible dosing timing to maximize adherence and tolerability.
20	Results of syntheses	For each synthesis, briefly summarise the characteristics and risk of bias among contributing studies. Present results of all statistical syntheses conducted. If meta-analysis was done, present for each the summary estimate and its precision and measures of statistical heterogeneity.	**Section 3.3** (*Synthesis of Evidence*), subsections 3.3.1–3.3.5. Narrative synthesis presented across five domains: (3.3.1) administration timing and day-of-week; (3.3.2) glycemic control; (3.3.3) weight loss outcomes; (3.3.4) adherence and patient satisfaction; (3.3.5) adverse events. No meta-analysis was performed due to heterogeneity of study designs.
21	Reporting biases	Present assessments of risk of reporting biases for each synthesis assessed.	**Section 4.4** (*Limitations*). Reporting bias is acknowledged: the review was limited to published literature, and no unpublished studies, conference abstracts, or grey literature were included. The narrow research question may have resulted in underrepresentation of relevant evidence on flexible dosing not explicitly mentioning day-of-week effects.
22	Certainty of evidence	Present assessments of certainty (or confidence) in the body of evidence for each outcome assessed.	**Sections 2.7 and 4.4**. The certainty of the body of evidence is discussed in the context of the quality assessment and limitations. The heterogeneous study designs, small number of included studies, and absence of direct comparative data on day-of-week effects substantially limit the certainty of evidence. This is explicitly acknowledged as a key limitation of the review.

Section 6 — DISCUSSION

Item	Section/Topic	Checklist Item	Location in Manuscript

No.			
23	Discussion	Provide a general interpretation of the results in the context of other evidence, and implications for future research.	**Section 4** (*Discussion*), subsections **4.1** (*Summary of Findings*), **4.2** (*Chronopharmacology and Clinical Implications*), **4.3** (*Implications for Clinical Practice*), and **4.5** (*Future Research Directions*). Results are interpreted in the context of the pharmacokinetic profiles of semaglutide and tirzepatide and the emerging field of chronopharmacology. Clinical implications for shared decision-making are discussed.
24	Limitations	Discuss any limitations of the evidence included in the review.	**Section 4.4** (*Limitations*). Five limitations are explicitly identified: (1) only three studies met inclusion criteria, none directly comparing days of the week; (2) heterogeneous study designs precluding meta-analysis; (3) no tirzepatide-specific timing studies identified; (4) grey literature, conference abstracts, and unpublished studies not included; (5) search strategy may not have captured all relevant studies on flexible dosing without explicit day-of-week terminology.
25	Conclusions	Provide a brief summary of the results and their implications in the context of the review question and other evidence, and suggest priorities for future research.	**Section 5** (*Conclusions*). The conclusions state that current evidence does not support a specific optimal day of the week for semaglutide or tirzepatide administration. Flexible dosing schedules tailored to individual patient preferences are recommended. Future prospective studies are identified as an urgent priority to systematically evaluate administration timing effects on clinical outcomes in real-world settings.

Section 7 — OTHER INFORMATION

Item No.	Section/Topic	Checklist Item	Location in Manuscript
26	Funding	Provide information about the funding for the review and the role of the funders in the review.	**Funding section** (after Author Contributions). Explicit statement: *"This research received no external funding."* No role of funders to declare.
27	Competing	Declare any competing	**Conflicts of Interest section** (after

interests	interests of review authors.	Funding). Explicit statement: *"The authors declare no conflicts of interest."*

PRISMA Compliance Statement (for inclusion in Methods)

> *"This systematic review was conducted in accordance with the Preferred Reporting Items for Systematic Reviews and Meta-Analyses (PRISMA) 2020 guidelines [Page MJ et al., BMJ 2021;372:n71]. The review protocol was not registered prospectively."*
>
> — Section 2.1, *Protocol and Registration*

PRISMA 2020 Flow Diagram Data Summary

Stage	Count
Records identified: SciSpace Deep Search	230
Records identified: SciSpace Basic Search	200
Records identified: SciSpace Full Text Search	300
Records identified: Google Scholar	219
Records identified: PubMed	522
**Total records identified**	**1,471**
Duplicates removed	471
Trimmed for feasibility	—
**Records screened (after deduplication)**	**1,000**
Records excluded at abstract screening	997
— Did not analyse administration timing/day-of-week effects	850
— General efficacy only (no temporal analysis)	100
— Focused on other GLP-1 RAs only	47
**Reports sought for retrieval**	**3**
Reports not retrieved	0
**Reports assessed for eligibility (full-text)**	**3**
Reports excluded at full-text screening	0
**Studies included in qualitative synthesis**	**3**

The PRISMA 2020 flow diagram (Figure 1) is included in the main text of the manuscript (Section 3.1).

End of PRISMA 2020 Checklist Response
